# A pragmatic clinical trial of hearing screening in primary care clinics: cost-effectiveness of hearing screening

**DOI:** 10.1186/s12962-022-00360-5

**Published:** 2022-06-25

**Authors:** Judy R. Dubno, Pranab Majumder, Janet Prvu Bettger, Rowena J. Dolor, Victoria Eifert, Howard W. Francis, Carl F. Pieper, Kristine A. Schulz, Mina Silberberg, Sherri L. Smith, Amy R. Walker, David L. Witsell, Debara L. Tucci

**Affiliations:** 1 Department of Otolaryngology-Head and Neck Surgery, Medical University of South Carolina, Charleston, SC USA; 2 Fuqua School of Business, Duke University, Durham, NC USA; 3 Department of Orthopaedic Surgery, Duke University School of Medicine, Durham, NC USA; 4 Department of Head and Neck Surgery & Communication Sciences, Duke University School of Medicine, Durham, NC USA; 5 Department of Medicine, Duke University School of Medicine, Durham, NC USA; 6 Center for Study of Aging and Human Development, Duke University School of Medicine, Durham, NC USA; 7 Department of Biostatistics and Bioinformatics, Duke University Medical Center, Durham, NC USA; 8 Department of Family Medicine and Community Health, Duke University School of Medicine, Durham, NC USA; 9 Department of Population Health Sciences, Duke University School of Medicine, Durham, NC USA; 10 National Institute On Deafness and Other Communication Disorders, National Institutes of Health, Bethesda, MD USA

**Keywords:** Hearing screening, Primary care clinic, Cost-effectiveness, Hearing loss, Older adults

## Abstract

**Background:**

Hearing loss is a high prevalence condition among older adults, is associated with higher-than-average risk for poor health outcomes and quality of life, and is a public health concern to individuals, families, communities, professionals, governments, and policy makers. Although low-cost hearing screening (HS) is widely available, most older adults are not asked about hearing during health care visits. A promising approach to addressing unmet needs in hearing health care is HS in primary care (PC) clinics; most PC providers (PCPs) do not inquire about hearing loss. However, no cost assessment of HS in community PC settings has been conducted in the United States. Thus, this study conducted a cost-effectiveness analysis of HS using results from a pragmatic clinic trial that compared three HS protocols that differed in the level of support and encouragement provided by the PC office and the PCPs to older adults during their routine visits. Two protocols included HS at home (one with PCP encouragement and one without) and one protocol included HS in the PC office.

**Methods:**

Direct costs of the HS included costs of: (1) educational materials about hearing loss, (2) PCP educational and encouragement time, and (3) access to the HS system. Indirect costs for in-office HS included cost of space and minimal staff time. Costs were tracked and modeled for each phase of care during and following the HS, including completion of a diagnostic assessment and follow-up with the recommended treatment plan.

**Results:**

The cost-effectiveness analysis showed that the average cost per patient is highest in the patient group who completed the HS during their clinic visit, but the average cost per patient who failed the HS is by far the lowest in that group, due to the higher failure rate, that is, rate of identification of patients with suspected hearing loss. Estimated benefits of HS in terms of improvements in quality of life were also far greater when patients completed the HS during their clinic visit.

**Conclusions:**

Providing HS to older adults during their PC visit is cost-effective and accrues greater estimated benefits in terms of improved quality of life.

*Trial registration*: clinicaltrials.gov (Registration Identification Number: NCT02928107).

## Introduction

Hearing loss is a high prevalence condition among older adults and increases as the population ages. However, most older adults are not asked about their hearing during health care visits, are not regularly screened for hearing loss, and have not had their hearing tested [22]. Several well-known factors also contribute to substantial unmet needs for hearing health care for older adults. First, many older adults wait many years to seek help after becoming aware (by themselves or by their communication partners) of hearing difficulties [8]. Second, only 15–20% of older adults who might benefit from hearing aids use them [5]. Third, those older adults who do acquire hearing aids delay their decision an average of 9 years after being identified as hearing-aid candidates [25]. These factors are a public health concern, given that hearing loss in older adults may be associated with higher risk for health co-morbidities, poorer health outcomes, poorer quality of life, and higher health care costs [23] (see Ref. [2] and [12] for reviews). Taken together, hearing loss in older adults results in high economic and social burden and is a societal concern to individuals and their families, communities, professionals, governments, and policy makers.

One promising approach that could help to alleviate this burden and address unmet needs in hearing health care is availability of low-cost hearing screening (HS) for older adults. In addition to the identification of individuals who may have hearing loss and are in need of diagnosis and treatment, a benefit of HS is increasing awareness and understanding of hearing loss, which may encourage adults who fail the HS to make other accommodations, such as changes in their communication strategies, listening environments, and use of assistive listening devices. Consistent with these benefits, the World Health Organization recommends hearing screening for older adults in their recent implementation handbook for hearing screening [36]. However, the US Preventative Services Task Force (USPSTF) recently reaffirmed their recommendation that, for asymptomatic adults 50 years or older, “the current evidence is insufficient to assess the balance of benefits and harms of screening for hearing loss in older adults” [14, p. 1196, 10, 21]. The USPSTF concluded that adequate evidence is available that screening tools, such as pure-tone detection measures, speech-in-noise tasks, or self-reported hearing questionnaires, *can* detect hearing loss in asymptomatic adults age 50 years and older. However, they also concluded that evidence is lacking that HS in older adults, and interventions to treat hearing loss in screened adults, ultimately improves functional hearing abilities, quality of life, and health outcomes.

This topic was previously addressed in a randomized clinical trial comparing no screening to two HS methods (hand-held otoscope, hearing handicap questionnaire) individually and in combination in a large sample of veterans ≥ 50 years of age [15, 38]. Patients who failed the screening were recommended for a diagnostic hearing assessment. The primary outcome measure was hearing aid use after one year in screened vs. unscreened groups. Depending on screening method, 19–64% failed the HS. Of those, 15–27% followed up with hearing assessments and 4–7% acquired hearing aids, these rates were significantly higher than for patients who were not screened. Although results were promising, this study was limited by a predominantly veteran population of men who are eligible for hearing services and hearing aids at no cost, and by recruitment methods that may have favored patients who had prior concerns about their hearing. An additional limitation was a primary outcome that was limited to hearing-aid use. Nevertheless, this study demonstrated the feasibility of widespread administration of HS in a busy health care setting, and the value of clinical trials in assessing effectiveness of HS in older adults.

A more recent randomized control trial of 1665 veterans and members of the community at several US locations [11] compared three HS methods (automated audiogram with results explained, four-frequency pure-tone HS with pass/fail results, a digits-in-noise HS with pass/fail results) to a control group that received no HS with half of each group also watching a 2-min educational video about hearing and hearing loss causes. Follow-up for reassessment with two self-reported hearing questionnaires occurred 6–8 months after screening, in addition to contact by the study team to determine if patients sought or received diagnostic assessments or hearing loss treatment. Follow-up was significantly greater in the four-frequency screener only versus the control group (24.6% vs.16.8%) and, for all groups, follow-up rates were higher for patients who failed than for those who passed the HS (27.2% vs. 4.7%).

Outcomes of widely available HS have also been studied in Australia using a telephone-based HS followed by a telephone interview several months later with people who failed the screening [17]. Of those, 36% subsequently followed up with a hearing health care professional, 13% of whom received recommendations to acquire hearing aids, and ~ 6.5% of whom followed this advice.

Another option that has been considered for facilitating provision of HS to large numbers of older adults is incorporating HS into routine primary care (PC) visits (such as the “Welcome to Medicare” visit and Medicare Annual Wellness visit). Improved uptake of HS in PC offices is already recognized as an important objective for PC, but this option is limited by perceived lack of clinician time [37], available resources, and skilled personnel to administer the HS. For example, Zazove et al. [40] used an alert in the electronic health record to prompt PCPs to conduct a single-question HS during the visit, which significantly increased referrals for and completion of diagnostic assessments. Although hearing and balance problems significantly affect older patients seen in PC offices, PCPs do not regularly inquire about hearing loss [7, 16, 31, 39]. One reason may relate to inadequate reimbursement for HS and hearing health care services, which then further reduces the likelihood of identifying patients with hearing loss. Despite this, to our knowledge, no assessment of cost of HS in community PC settings has been conducted in the US [3]. Morris [19] and Morris et al. [20] used Markov models to demonstrate that screening all adults at age 60, 65, and 70 years for bilateral hearing loss ≥ 35 dB HL is cost effective in the UK as compared to no screening, similar results were found for 50–70-year-old adults in the Netherlands [18]. A recent scoping review concluded that, regardless of method, HS of older adults is cost-effective compared to no HS [12].

Several HS methods are in use (see Ref. [6] and [10] for reviews), but use in the PC office setting may be limited by cost and maintenance of instrumentation, time or resource intensity, and low sensitivity and specificity. Telephone-based, self-administered triple-digit HS, based on the procedure developed by Smits et al. [27] and modified for use in the US by Watson et al. [32], utilizes digits in noise to minimize effects of background noise in the test environment and simple, telephone-keypad patient responses. These features, combined with reported good sensitivity and specificity (0.80 and 0.83 respectively), using pure-tone average > 20 dB HL as the criterion measure [32, 35] make the digits-in-noise test an ideal HS procedure to administer in a patient’s home or in the PC office. The triple-digit HS procedure was also appropriate to the primary outcome measure of the clinical trial (percent of patients in each group who completed the HS within 120 days of the PCP visit). Implementation of a HS procedure that is not resource intensive but is highly sensitive and specific would allow PCPs to advocate for a first step in providing hearing health care. As the PCP is a major source of help to those seeking care after failure of HS, it is possible that active encouragement by the PCP to perform and follow through on the HS would positively affect outcomes.

### Practice-level costs of care

To change PCP and reimbursement practices, identification of optimal strategies that balance PC practice efficiency (and cost) with effective hearing health care delivery is necessary. Approaches will vary by the level and type of PCP involvement that is most important in improving hearing health outcomes and we need to determine what costs are justified by outcomes. Knowledge of health care operations and cost are needed to address gaps in understanding of practice-level costs and cost effectiveness.

### Purpose of the current study

The purpose of this study was to estimate the cost effectiveness of three HS protocols that differed in the level of support by the PC office and the PCPs using data from a recently conducted pragmatic clinic trial (see Ref. [1, 26] for more details). HS consisted of the self-administered telephone-based digits-in-noise test as mentioned earlier [32]. Patients who were 65–75 years of age and scheduled for routine visits at one of six PC practices in the Durham, NC area and with no prior history of a hearing loss diagnosis or hearing-aid use were invited to enroll in the trial. All enrolled patients received written information about hearing loss warning signs, consequences of hearing loss on communication, and interventions for hearing loss, as well as printed instructions about how to complete the telephone-based HS. Three protocols were employed, with two clinics agreeing to one of three protocols. Patients were then recruited from each of the six participating clinics. The protocols differed in the setting of the HS (at home or in-clinic) and whether (yes/no) the PCP encouraged home-based HS. Thus, three protocols differed in the level of clinical and patient support, as follows: (1) no PCP encouragement to complete the HS at home (Home-NPE), (2) PCP encouragement to complete the HS at home (Home-PE), and (3) PCP encouragement and opportunity to complete the HS in the PC office during the patient’s office visit with space provided for the HS (Clinic-PE). The primary outcome was the percent of patients in each group who completed the HS within 120 days of the PCP visit.

All patients received results at the end of the HS; patients who failed were contacted by the study coordinator either in the PC office (Clinic-PE) or by telephone within one week (Home-NPE and Home-PE) and offered an appointment for a diagnostic assessment and medical evaluation at the Duke Otolaryngology-Head and Neck Surgery clinic. Secondary outcomes included the number of patients in each group who (1) failed the HS, and, of those who (2) scheduled and (3) completed the diagnostic assessment within 120 days of the HS, and, of those who (4) received a hearing loss intervention plan from a hearing healthcare provider (otologist and/or audiologist).

Direct costs of the HS were determined by (1) cost of the printed educational materials about hearing loss, (2) cost of PCP time to educate the patient, estimated to be < 3 min, (3) cost of providing PCP encouragement for at-home HS, (4) cost of providing PCP encouragement for in-clinic HS, and (5) cost of access to the HS telephone system (same for at-home or in-clinic). Indirect costs for the PC office screening include cost of space and minimal staff time to direct the patient to the room for HS. Costs were tracked and modeled for each phase of care during and following the HS, including completion of a diagnostic assessment and follow-up with the recommended treatment plan.

### The patient path

The pragmatic clinical trial enrolled 220 eligible patients in each of the three groups (a total of 660 patients). This required screening 955 patients for eligibility. To estimate costs, the entire series of patient exit points (“off-ramps”) from the trial were considered, beginning from when each patient was approached in a PC office about their interest and eligibility for participation, to the endpoint when a small number of patients chose to acquire hearing aids as recommended by a hearing health care provider. This patient path through the pragmatic clinical trial is illustrated in Table 1, along with the numbers of patients in the three groups in the trial at each step in the path, notations, and related costs.

**Table 1:**
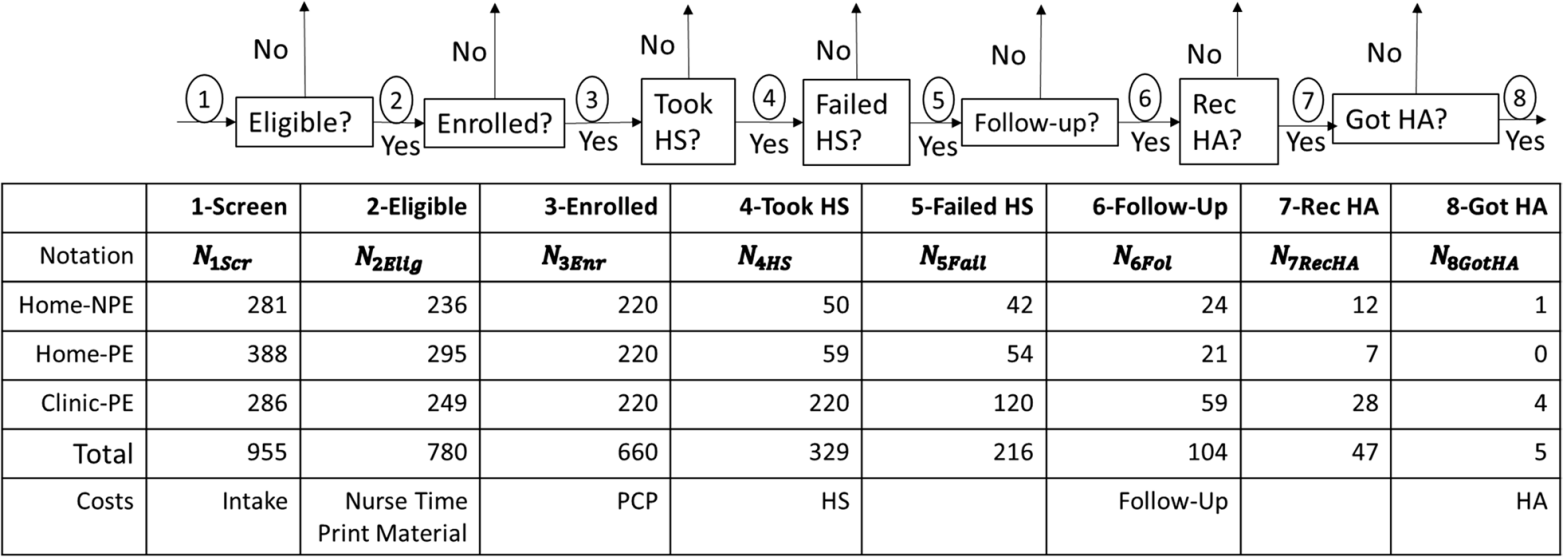
The patient path, associated cost categories, and exit points (“off-ramps”) for three patient groups

The patient path began with each of the 955 patients being asked a series of screening questions to determine eligibility (Step 1 in Table 1). A total of 175 patients were found to be ineligible and, by far, the most common reason for ineligibility was prior history of hearing loss (94.8%). After eligibility was determined, each of the 780 eligible patients was given educational materials about the trial and asked if they would like to enroll (Step 2); 120 patients declined, leaving a total of 660 enrolled patients (Step 3). The two most common reasons for declining were not being interested in participating (34.2%) and denial of hearing problems (30.8%). At Step 4, the number of enrolled patients who completed the HS within the required time was determined (329). Step 5 determined the number of patients who completed the HS and failed (216). Step 6 determined the number of patients who failed the screening and completed a diagnostic assessment (104). Step 7 determined if, at that diagnostic assessment, one or both of their hearing health care providers recommended hearing aids (47). Finally, Step 8 determined the number of those patients who acquired hearing aids (5).

When comparing the number of patients at each step across the three groups (Table 1), we observed that the Home-PE group required screening a higher number of patients for eligibility and a higher number of eligible patients declined to be enrolled, as compared to the Home-NPE and Clinic-PE groups. Upon further examination, there did not seem to be any significant difference between the cohorts in terms of observable demographic or age profiles; thus, we assume that these differences may be due to other unobserved population characteristics, or other random factors.

As is common to many other similar studies, the final number of patients who remained on the entire path of the trial is exceedingly small (Step 8). Even in the Clinic-PE group, where all 220 enrolled patients completed the HS, only four patients obtained hearing aids following their providers’ recommendations, although this was the largest number of the three groups. This outcome is consistent with evidence that people wait years before taking action after learning they have hearing loss (e.g., [25]). Notably, the largest number of patients exiting the trial occurred at the decision step of completing or not completing the HS (Step 4), followed by the number of patients who failed the HS who did or did not schedule and complete a diagnostic assessment (Step 6).

## Methods

### Cost parameters from the clinical trial

Along with the data from the clinical trial, Table 1 also lists the notation used in calculating costs and benefits. The variable N was used to denote the number of patients, along with a subscript to indicate the count at a specific step of the process, as described. Thus, for example, ${N}_{3Enr}$ with the subscript “3Enr” denotes Step 3 in the off-ramp diagram (Table 1), which was the number of patients who were enrolled. The specific patient group being described (e.g., the Home-PE group, or other groups) is expected to be evident from the context. In case of any ambiguity, we used superscripts to clarify the group (e.g., ${N}_{3Enr}^{Home-PE}$) in our analytic process, but we have chosen to not include these superscripts herein to improve readability.

Next, the parameters that were used to estimate the costs of the process are listed in Table 2. Most of these are not empirical values, but best estimates of the relevant costs, which can differ across provider settings.

The follow-up (diagnostic assessment) cost was obtained from the Duke University Medical Center, using data from 2018. Observe that the data available lists the costs for a hospital-based clinic visit, as well as for a private clinic visit (i.e., a non-hospital setting, usually a stand-alone clinic). Within these two settings, there is a retail cost, as well as a discounted rate for research. The hospital-based retail cost was $1,206, whereas the discounted research cost was $308. The private clinic retail cost was $422 and the research cost was $136. We have chosen to use the private clinic retail cost for our follow-up cost figure for diagnostic assessment, and the reader is free to use the other figures if they deem them relevant to their own context. The hearing-aid cost was obtained from Strom [28] and represents an average cost for two (bilateral) hearing aids.

To translate these cost rates into the actual cost per patient, we estimate the time taken for each resource that has an hourly cost. These time estimates are listed in Table 3.

**Table 2:** Estimated cost parameters

Description	Variable	Estimate
Intake cost per hour	$${C}_{F}$$	$20/h
Nurse cost per hour	$${C}_{N}$$	$50/h
PCP cost per hour	$${C}_{D}$$	$150/h
Printed material cost per unit	$${C}_{Print}$$	$0.05
HS cost per patient	$${C}_{Screen}$$	$8
Screening room cost per hour	$${C}_{Room}$$	$50/h
Follow-up cost	$${C}_{FollowUp}$$	$422
Hearing aid cost (bilateral)	$${C}_{HA}$$	$4600

Each of these costs was incurred at a different point in the patient path. Specifically, as the numbers of patients still on the path decreases, patients no longer in the trial do not incur the costs associated with the interaction with their providers and facilities. This is also shown in Table 1. For example, all patients incur the Intake cost, when patients are screened for eligibility. Thus, ${N}_{1Scr}$ patients incur the Intake cost. Next, only eligible patients incur the Nurse Time cost. Thus, ${N}_{2Elig}$ incur this cost, and so on. Note that the PCP time taken for each patient is slightly less in the Home-NPE group, based on the design of the clinical trial related to PCP encouragement.

## Results

### Calculations from the clinical trial

The first set of calculated values is the percentages of patients who were retained in the trial at different steps in the patient path (Table 4). For some values, there are three basis numbers used to calculate percentages—the screened patient count ${N}_{1Scr}$, the eligible patient count ${N}_{2Elig}$, and the enrolled patient count ${N}_{3Enr}$. Thus, the percentages listed in Table 4 are not exhaustive.

**Table 3:** Estimated resource usage per patient

Description	Variable	Home-NPE Estimate	Home-PE Estimate	Clinic-PE Estimate
Time taken at intake	${T}_{F}$	2 min	2 min	2 min
Time taken with the nurse	${T}_{N}$	2 min	2 min	2 min
Time taken with the PCP	${T}_{D}$	3 min	5 min	5 min
Time taken to complete HS	${T}_{Screen}$	10 min	10 min	10 min

Finally, these data provided the costs per patient in the three patient groups (Table 5). The time estimates have been converted to hours to match with hourly costs.

**Table 4:** Calculated percentages from the three patient groups

Description	Formula	Home-NPE	Home-PE	Clinic-PE
Percent of all patients who were eligible	$\frac{\left({N}_{1Scr}-{N}_{2Elig}\right)}{{N}_{1Scr}}$	84.0%	76.0%	87.1%
Percent of all patients who enrolled	$\frac{{N}_{3Enr}}{{N}_{1Scr}}$	78.3%	56.7%	76.9%
Percent of eligible patients who enrolled	$\frac{{N}_{3Enr}}{{N}_{2Elig}}$	93.2%	74.6%	88.4%
Percent of eligible patients who completed HS	$\frac{{N}_{4HS}}{{N}_{2Elig}}$	22.7%	26.8%	100.0%
Percent of patients who completed the HS who failed	$\frac{{N}_{5Fail}}{{N}_{4HS}}$	84.0%	91.5%	54.5%
Percentage of eligible patients who failed the HS	$\frac{{N}_{5Fail}}{{N}_{2Elig}}$	17.8%	18.3%	48.2%

Table 5 has four sections, which are separated visually by horizontal lines.

- The first section lists the six elements of cost that are incurred in the PCP visit. It converts hourly costs of various resources into a cost when a particular patient utilizes that resource or service.
- The second section combines these costs with the numbers of patients who utilize the respective resource or services to determine the total cost across all patients in that group. Note that the first four costs are incurred when the patient visits the PCP. The next two are the cost of follow-up diagnostic assessment (which is incurred in a subsequent visit with a different provider and is paid by the study) and the hearing-aid cost (which is incurred by the patient, their insurance, or other subsidy).
- The third section sums all PCP costs, followed by calculation of four per-patient costs. The first considers the average cost per eligible patient, the second considers the average cost per enrolled patient, the third considers the average cost per patient who completed the HS, and the fourth considers the average cost per patient who failed the HS. For this analysis, we focused on per-patient variable costs, rather than on fixed, up-front costs.
- Finally, the fourth section sums all the costs, including those after the PCP visit, namely costs of the follow-up diagnostic assessments (Step 6 in Table 1) and costs of hearing aids (Step 8 in Table 1). Once again, four different per-patient costs are calculated—the first considers the average cost per eligible patient, the second considers the average cost per enrolled patient, the third considers the average cost per patient who completed the HS, and the fourth considers the average cost per patient who failed the HS.

These calculations generate some important metrics. First, let us consider the cost analysis up to Step 4 in the patient path (number of patients who completed the HS and failed). The average PCP cost per eligible patient is the highest in the Clinic-PE group ($27.96). The average PCP cost for enrolled patients is similarly highest for the Clinic-PE group ($31.64). However, the average PCP cost per patient who failed the HS is highest in the Home-PE group ($73.83) and lowest in the Clinic-PE group ($58.01). The average cost per patient who failed the HS varies by about 20% across all three groups and ranged from about $58 to about $73. This cost varies from group to group because the number of patients who failed the HS in each group differs. This is discussed further in the next section.

Second, let us consider the cost analysis for the entire patient path. The average cost per eligible patient is $73.60 for the Home-NPE group, $43.56 for the Home-PE group, and $201.85 for the Clinic-PE group. The average cost per enrolled patient shows a similar profile- $78.96, $58.40, and $228.45, respectively. The average cost per patient who failed the HS is $413.58, $237.95, and $418.83, which shows that the metric for Home-NPE and for Clinic-PE are essentially the same.

### Patient identification rates

**Fig. 1:**
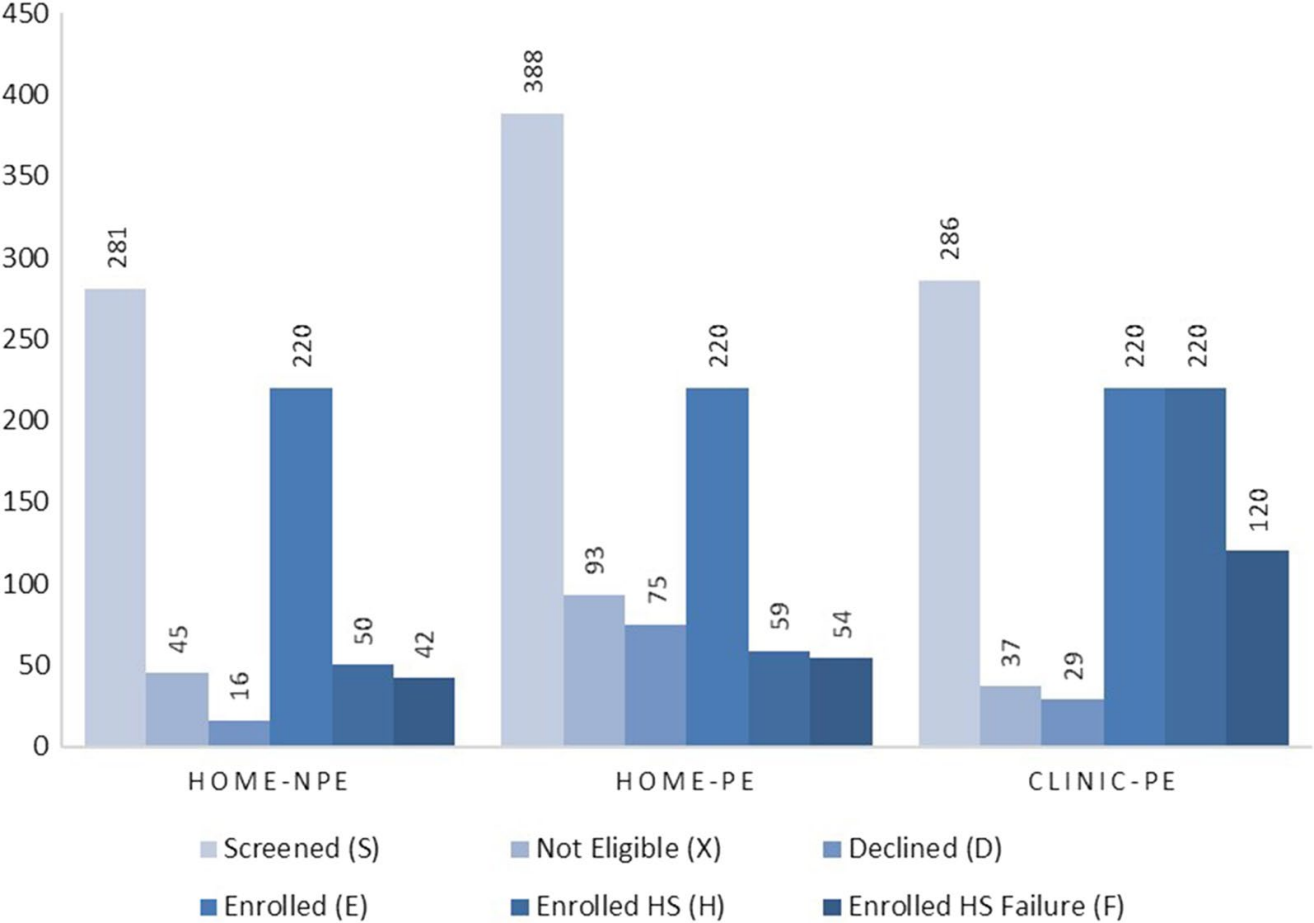
Selected data from the pragmatic clinical trial for three patient groups

The data from the clinical trial revealed some important behavioral and hearing health indications. These data are shown in Fig. 1.

First, Fig. 1 shows that there is only a small difference in the choices made by patients with or without PCP encouragement. The Home-NPE group completed the HS at a rate of 22.7% (50 of 220), whereas the Home-PE group did so at a rate of 26.8% (59 of 220) (see [26] for more details). Second, the availability of an in-clinic HS option led to fewer declines from patients (comparing the two cases with PCP encouragement). The Clinic-PE group results show 29 declines of 249 *eligible* patients (11.7%), whereas the Home-PE group results show 75 declines of 295 *eligible* patients (25.4%). The Home-NPE group does show fewer declines. Note that all *enrolled* patients in the Clinic-PE group completed the HS (220/220, 100%). Third, in both the Home-PE and Home-NPE groups, very large percentages of the patients who completed the HS failed. The numbers are 42 of 50 in the Home-NPE group (84.0%) and 54 of 59 in the Home-PE group (91.5%). These results suggest that when patients decide to complete the HS at home, those who make this decision almost certainly suspect they will fail the HS, which may mean that they are already concerned about their hearing and communication abilities. Fourth, the rate at which patients in the clinical trial cohort can be expected to fail the HS is probably closest to the rate obtained in the Clinic-PE group, because this is where 100% of the 220 patients completed the HS. This group shows an HS failure rate of 54.5%, which we can refer to as the “gold standard.”

Thus, asking patients to take the HS at home leads to very large numbers of patients who may have failed the HS but were not identified. This can be estimated as the difference in percentages between the Clinic-PE group and the other two groups combined. The Clinic-PE group HS failure rate is 120/220 = 54.5%, whereas the combined failure rate of the other two groups is (42 + 54)/440 = 21.8%. Thus, referring to the “gold standard” failure rate of 54.5%, the HS at home procedure leads to missed identification of almost one-third (32.7%) of patients with suspected hearing loss. It is possible that subsequent PCP visits and repeat opportunities for HS will identify some of these missed patients.

### Patient identification costs

Patient identification costs were generated from the results of the clinical trial reviewed earlier and summarized in Fig. 1. We present the cost component breakdown using two baseline numbers—the number of patients who *completed* the HS and the number of patients who *failed* the HS. It is important to consider both because there is value in completing the HS even for patients who pass.

**Fig. 2:**
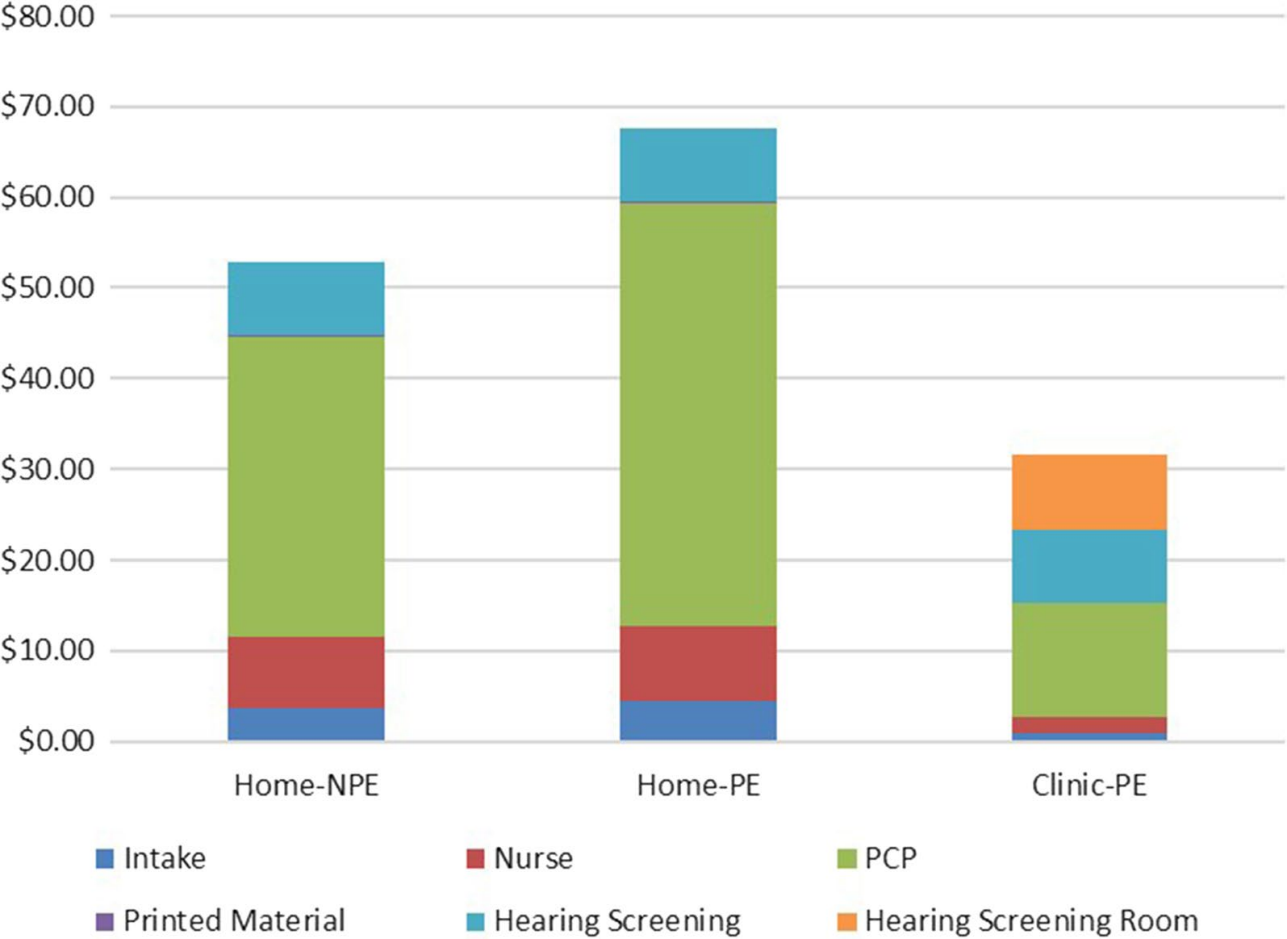
Per-patient cost components for each group for each patient who *completed* the HS

The per-patient cost components in each patient group for each patient who *completed* the HS are presented in Fig. 2. These costs were labelled $$\frac{{S}_{5Fail}}{{N}_{4HS}}$$, and are $52.85 for the Home-NPE group, $67.58 for the Home-PE group, and $31.64 for the Clinic-PE group, from Table 5.

**Fig. 3:**
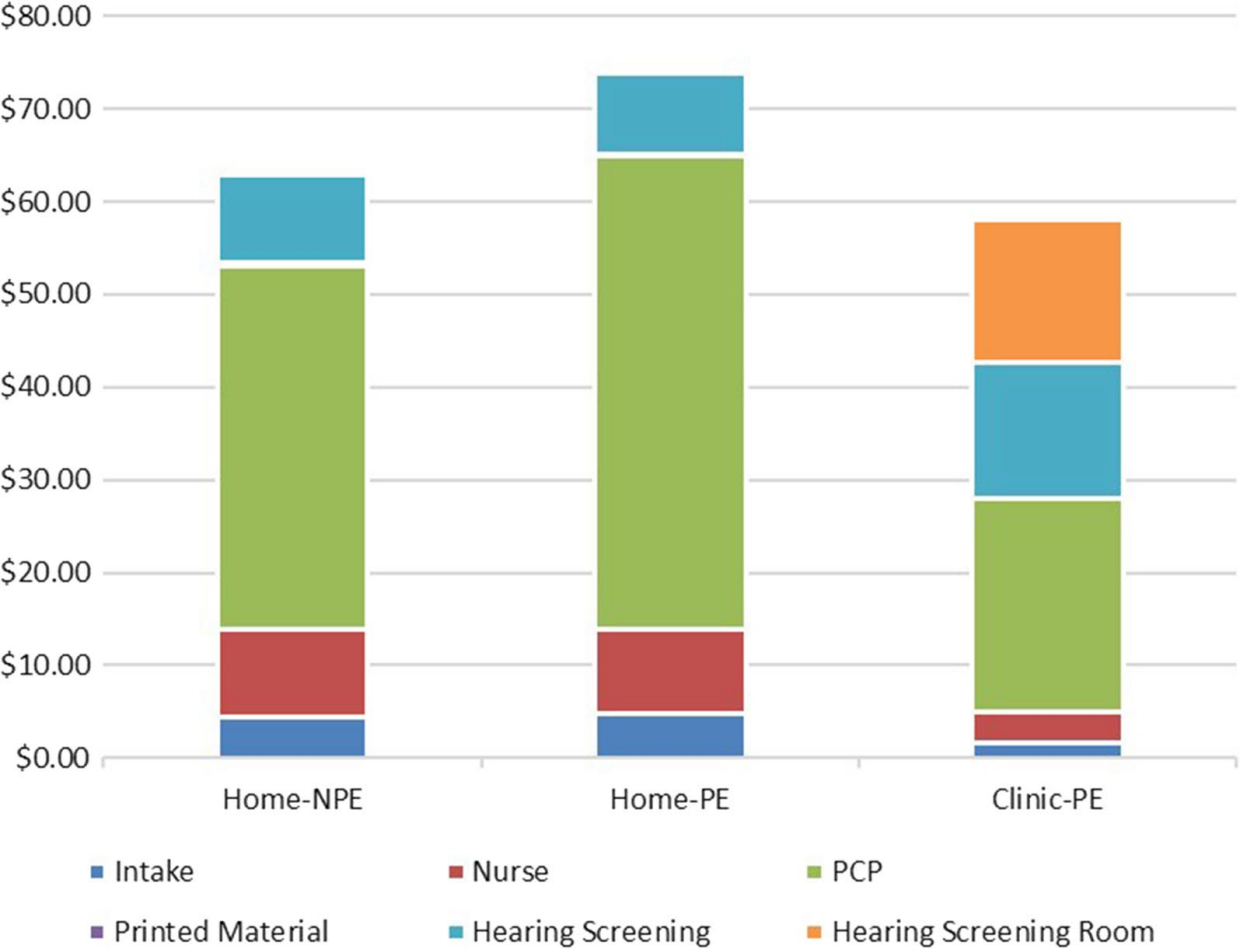
Per-patient cost components for each group for each patient who *failed* the HS

Next, we consider the per-patient cost components in each patient group for each patient who *failed* the HS. These costs were labelled $$\frac{{S}_{5Fail}}{{N}_{5Fail}}$$ and were $62.92 for the Home-NPE group, $73.83 for the Home-PE group, and $58.01 for the Clinic-PE group, from Table 5. These are shown in Fig. 3.

First, observe from Figs. 2 and 3 that some cost components are higher in the Home-PE and Home-NPE groups because some of the in-clinic activities must occur even if the patient chooses to not complete the HS at home. The large non-completion rates for these two groups lead to a higher final cost for each HS failure identified.

Second, as observed earlier, the encouragement from the PCP in the Home-PE group leads to only a very small change in HS completion rates (compared to the Home-NPE group). Because the PCP cost is the largest component, the additional time results in the Home-PE group being the costliest for each HS failure identified.

Third, in the Clinic-PE group, although the in-clinic HS room is a significant added cost, 100% completion rate and the much higher identification rate of HS failures means that, despite the added cost, this group has the lowest overall cost for each HS failure identified.

Thus, the cost results from this pragmatic clinical trial suggest that of the three protocols, the Clinic-PE meets the health needs of this 65–75-year-old patient population the best and is also the least costly in terms of the cost to identify each HS failure. Costs of HS are only one side of the cost-effectiveness analysis, and the full picture will develop once we consider the benefits of HS for each patient in each cohort, as discussed in the next section.

## Discussion

### Benefits of HS

For estimating the benefits of HS, it is necessary to make a series of critical theoretical assumptions. We also break out the benefits based on the assumptions so that readers may consider the imputed benefits based on their own assessments of these assumptions.

There are some benefits for patients at each step of the patient path (Fig. 1), even considering the various exit points, or off-ramps. We have considered five exit points where we may assign benefits to the patients. We assume that these benefits are captured through an estimation of an improvement in their quality of life, and thus we use quality-adjusted life years (QALYs), which is a widely used health economic metric (see Ref. [33] and [34] for reviews) and has been generally accepted for use as a health outcome measure in studies of cost-effectiveness of hearing healthcare [3, 4, 13]. If a patient is aware of their condition, and takes actions to remedy or address this condition, then this leads to an incremental improvement in their quality of life. Thus, QALY is an index that provides a way to compare the value and benefits of interventions across health conditions (and in this case, across trial exit points) and uses the same metric (dollars) as was used to estimate costs. In order to compare QALYs and costs of intervention, we use the monetary value of a QALY improvement, which has been estimated at between $50,000-$150,000 (e.g., [24, 30]). Here, we will use an estimate of $100,000 for a QALY improvement. Using the monetary value of a QALY improvement allows health care providers and policy makers to compare (in dollars) the cost of an investment in a screening program or a treatment and the resulting benefit to public health.

Recall that the clinical trial identified patients who were between 65 and 75 years of age and were visiting their PCP for a routine visit. These patients may have hearing difficulties but have not yet been identified or advised to address their hearing loss. Accordingly, we started with the estimated benefits to the patients who remained in the trial for the entire patient path, that is, were identified as failing the HS, followed up with a diagnostic assessment, received an intervention plan, and acquired hearing aids. Thus, the long-term benefit of identifying such patients through HS is to provide improved communication and better quality of life (and presumably better health outcomes) during the years they would not have otherwise been identified as having hearing loss.

We assume that at some point after 65 years of age all patients will address any potential hearing loss, which may be well after their hearing loss has progressed. However, this is not uniformly the case, because there are few national guidelines regarding universal HS for older adults (e.g., [14]). Thus, many older adults may have poorer quality of life due to undetected and untreated hearing loss. We can further assume that hearing loss will be detected eventually as hearing difficulties increase in a patient, and that the HS intervention will identify such loss on average two years earlier for each patient. This is consistent with evidence that the average age of first-time hearing aid users is ~ 70 years of age [8] and that individuals who had spoken with a physician about their hearing difficulties were more likely to have had a hearing assessment in the recent past [22]. We also assume that the earlier detection improves each patient’s quality of life by an average of 0.1 QALY each year. This is consistent with [13], and with the 10% disability rating for hearing impairment provided by the Veterans Benefit Administration, as detailed in VA Benefits and Health Care, 38 CFR Book C, Schedule for Rating Disabilities [29]. Thus, the benefit for each such patient is a total of 0.2 QALY. We also provide estimates in a later section using a smaller assumption of the QALY benefit to a patient.

Using 0.2 as the maximum QALY improvement possible, we make the following assumptions, starting from the patient group who accrued the most benefit (those who acquired hearing aids).

- A patient who received a recommendation to acquire hearing aids but did not follow the recommendation still accrues the benefit of receiving that recommendation and any other information and advice from their hearing healthcare provider. This may lead the patient to be vigilant and follow the recommendation later, but earlier than may have happened without this intervention. Each such patient is estimated to accrue a 0.1 QALY benefit.
- A patient who followed up with a diagnostic assessment with a hearing healthcare provider and was not recommended to acquire hearing aids still likely receives important information from their provider about hearing loss awareness, prevention, and improved communication strategies, and is therefore estimated to accrue a benefit of 0.075 QALY.
- A patient who failed the HS and did not follow up with a diagnostic assessment is estimated to accrue a benefit of 0.05 QALY, based on the failure information provided by the HS and the printed educational materials provided earlier in the patient path.
- Finally, a patient who completed the HS and passed is estimated to accrue a benefit of 0.025 QALY, for the same reasons outlined for patients who failed the HS.

These estimated QALY benefits to the three patient groups are summarized in Table 6, along with the number of patients who remained in the study to that step in the patient path. This is used to determine the aggregate QALY benefit to all patients in each of the groups. The first row enumerates our estimated benefits; the next three rows list the number of patients who completed each step, and the final three rows show the total QALY benefits across each group.

**Table 5:** Cost calculations for patients in each group

Description	Formula	Home-NPE	Home-PE	Clinic-PE
Intake costs	$$\frac{{T}_{F}}{60}.{C}_{F}$$	$0.67	$0.67	$0.67
Nurse costs	$$\frac{{T}_{N}}{60}.{C}_{N}$$	$1.67	$1.67	$1.67
PCP costs	$$\frac{{T}_{D}}{60}.{C}_{D}$$	$7.50	$12.50	$12.50
Printed material cost	$${C}_{Print}$$	$0.05	$0.05	$0.05
HS cost	$${C}_{Screen}$$	$8.00	$8.00	$8.00
HS room cost	$$\frac{{T}_{Screen}}{60}.{C}_{Room}$$	–	–	$8.33
Total intake cost	$$\left(\frac{{T}_{F}}{60}.{C}_{F}\right).{N}_{1Scr}$$	$187.33	$258.67	$190.67
Total nurse cost and printed material cost	$$\left(\frac{{T}_{N}}{60}.{C}_{N}+{C}_{Print}\right). {N}_{2Elig}$$	$405.13	$506.42	$427.45
Total PCP cost	$$\left(\frac{{T}_{D}}{60}.{C}_{D}\right). {N}_{3Enr}$$	$1650.00	$2750.00	$2750.00
Total HS and screening room (if applicable) cost	$$\left({C}_{Screen}+\frac{{T}_{Screen}}{60}.{C}_{Room}\right).{N}_{4HS}$$	$400.00	$472.00	$3593.33
Total follow-up cost	$${C}_{FollowUp}.{N}_{6Fol}$$	$10,128.00	$8,862.00	$24,898.00
Total hearing aid cost	$${C}_{HA}.{N}_{8GotHA}$$	$4600.00	$0.00	$18,400.00
Sum of clinic costs (i.e., until HS failure)	$${S}_{5Fail}=\left(\frac{{T}_{F}}{60}.{C}_{F}\right).{N}_{1Scr}+\left(\frac{{T}_{N}}{60}.{C}_{N}+{C}_{Print}\right). {N}_{2Elig}+\left(\frac{{T}_{D}}{60}.{C}_{D}\right). {N}_{3Enr}+\left({C}_{Screen}+\frac{{T}_{Screen}}{60}.{C}_{Room}\right).{N}_{4HS}$$	$2642.47	$3987.08	$6961.45
Average PCP cost per eligible patient	$$\frac{{S}_{5Fail}}{{N}_{2Elig}}$$	$11.20	$13.51	$27.96
Average PCP cost per enrolled patient	$$\frac{{S}_{5Fail}}{{N}_{3Enr}}$$	$12.01	$18.12	$31.64
Average PCP cost per patient who completed HS	$$\frac{{S}_{5Fail}}{{N}_{4HS}}$$	$52.85	$67.58	$31.64
Average PCP cost per patient who failed HS	$$\frac{{S}_{5Fail}}{{N}_{5Fail}}$$	$62.92	$73.83	$58.01
Sum of all costs	$$S={S}_{5Fail}+{C}_{FollowUp}.{N}_{6Fol}+{C}_{HA}.{N}_{8GotHA}$$	$17,370.47	$12,849.08	$50,259.45
Average cost per eligible patient	$$\frac{S}{{N}_{2Elig}}$$	$73.60	$43.56	$201.85
Average cost per enrolled patient	$$\frac{S}{{N}_{3Enr}}$$	$78.96	$58.40	$228.45
Average cost per patient who completed HS	$$\frac{S}{{N}_{4HS}}$$	$347.41	$217.78	$228.45
Average cost per patient who failed HS	$$\frac{S}{{N}_{5Fail}}$$	$413.58	$237.95	$418.83

**Table 6:**
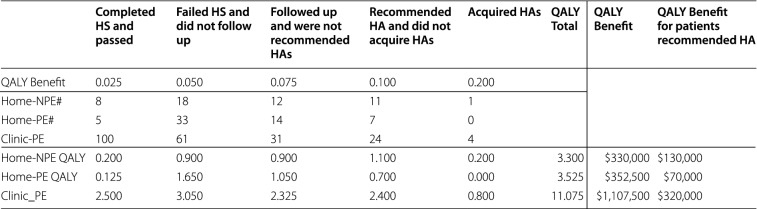
HS outcomes and benefits

Thus, from these assumptions we estimate that the net QALY benefit to the Home-NPE group, the Home-PE group, and the Clinic-PE group are 3.300 QALY, 3.525 QALY, and 11.075 QALY, respectively. Using the figure of $100,000 per QALY from the literature, the benefits to each group are $330,000, $352,500, and $1,107,500 respectively, which are much greater than the total costs of $17,370, $12,849, and $50,259 for each group listed in Table 5. Even if we restrict the cost analysis to patients who followed up with a diagnostic assessment and were recommended hearing aids (including patients who did not acquire hearing aids and those who did), the QALY benefits are $130,000, $70,000, and $320,000, respectively (e.g., for the Home-NPE group, benefits are 1.1 + 0.2 QALY from Table 6).

We may also calculate these QALY benefit estimates for more conservative assumptions, namely where we reduce the QALY benefit assumptions for each patient by 50% (e.g. the total benefit to a patient who acquired a HA is now 0.1 QALY instead of 0.2, and so on). This leads to benefits to each group of $165,000, $176,250 and $553,750 (predictably, these are 50% of the benefit figures in the previous paragraph). These benefit figures are still much higher than the costs for each group.

These estimates also show that, by far, the greatest benefits to patients are in the Clinic-PE arm of the trial, at a slightly higher cost. These costs are dwarfed by the identified benefit from the improvement in the quality of patients’ lives. This is an overwhelming argument in favor of routinely including HS in PC offices for all older adults.

## Summary and conclusions

In this study, we estimated the cost-effectiveness of three HS protocols that differed in the level of support by the PC office and the PCPs. A total of 660 patients, 65–75 years of age, were enrolled in one of the three protocols during their routine PCP appointments; patients had no prior history of a hearing loss diagnosis or hearing-aid use. All enrolled patients received written information about hearing loss and printed instructions about how to complete the HS. The primary outcome was the percent of patients in each protocol who completed the HS within 120 days of the primary care visit. Patients learned the results of the HS and those who failed were invited to schedule and complete a diagnostic assessment and receive an intervention plan from a hearing healthcare provider. To estimate the cost-effectiveness of each HS protocol, costs included those of the printed materials, staff time, PCP time to educate the patient and provide encouragement for at-home or in-clinic HS, access to the HS, and clinic space for the HS (as applicable). Costs were tracked and modeled for each point on the patient path before, during, and after the HS.

For patients who completed the HS at home, very large percentages of patients failed, likely because they were already concerned about their hearing. It is also possible that a higher failure rate when completing the HS at home was due to a poorer home testing environment, which we are unable to confirm. Nevertheless, the HS procedure is conducted in a background of noise, so a very quiet environment is not required. Regardless of the reason, completing the HS at home lead to almost one-third of patients with suspected hearing loss who remain unidentified. In contrast, for the 100% of patients who completed the HS during their PC clinic visit, only about half of the patients failed, which is likely the true failure rate for this older adult patient sample. The cost-effectiveness analysis showed that the average PCP cost per eligible or enrolled patient is highest in the patient group who completed the HS during their clinic visit, but the average PCP cost per patient *who failed the HS* is by far the lowest in that group. Similar results were found for the average cost for the entire patient path per eligible or enrolled patient for those who failed the HS. For this patient group, although providing a room for the HS is an added cost, the higher rate of identification of patients with suspected hearing loss means that HS in the clinic setting has the lowest overall cost for each HS failure identified.

Finally, the benefits of HS were captured by estimating improvements in quality of life through the QALY metric; the net QALY benefit was compared for the three protocols. The estimates revealed that, by far, the greatest benefit was accrued when patients completed the HS during their clinic visit, despite a slightly higher cost, which provides substantial support for making HS available to older adult patients during their routine PCP visits. Future studies may consider other cost-effective solutions during PCP visits to increase HS completion rates, such as HS methods that do not require telephones or quiet rooms (such as self-report questionnaires) and electronic health records alerts to clinic personnel to ask patients about their hearing [9, 40]. Further research in cost-effectiveness of HS is also needed to test assumptions related to the QALY benefit estimates.

## Data Availability

The datasets used and/or analyzed during the current study are available from the corresponding author on reasonable request.
